# Heterogeneous effects of sleep and psychological health on pregnancy outcomes in donor insemination: a prospective cohort study

**DOI:** 10.3389/fpubh.2026.1821081

**Published:** 2026-08-26

**Authors:** Changsha Yin, Yan Wang, Yan Pu, Yuyang Wang, Biru Luo

**Affiliations:** 1 Department of Reproductive Medicine Nursing, West China Second University Hospital, Sichuan University, Sichuan University, Chengdu, China; 2 Key Laboratory of Birth Defects and Related Diseases of Women and Children (Sichuan University), Ministry of Education, Chengdu, Sichuan, China; 3 Department of Nursing, West China Second University Hospital, Sichuan University, Sichuan University, Chengdu, China

**Keywords:** anxiety, assisted reproductive technology (ART), depressive symptoms, donor insemination, IUI-D, IVF/ICSI-D, pregnancy outcomes, prospective cohort study

## Abstract

**Objective:**

To investigate the impact of sleep quality, anxiety, depression and somatic symptom burden on pregnancy outcomes among patients receiving donor-sperm assisted reproduction, and to explore heterogeneous associations across IVF/ICSI-D and IUI-D subgroups.

**Methods:**

A prospective cohort study was conducted at the Reproductive Center of West China Second University Hospital, Sichuan University. From June 2020 to May 2024, 758 consecutive patients undergoing donor insemination-assisted reproduction were enrolled, including 234 undergoing IVF/ICSI-D and 516 undergoing IUI-D. Somatic symptom burden, anxiety, depression, and sleep quality were assessed using the Patient Health Questionnaire-15 (PHQ-15), Generalized Anxiety Disorder-7 (GAD-7), Patient Health Questionnaire-9 (PHQ-9), and Pittsburgh Sleep Quality Index (PSQI), respectively. Baseline demographic characteristics and infertility-related variables were systematically recorded. Univariate screening followed by fully adjusted multivariable logistic regression and sensitivity analysis excluding educational attainment were performed to identify independent predictors of multiple pregnancy endpoints.

**Results:**

In the IVF/ICSI-D subgroup, higher PSQI scores (indicating poorer sleep) displayed independent correlative links to lower odds of clinical pregnancy (OR = 0.827, *p* = 0.012) and live birth (OR = 0.850, *p* = 0.031); higher sleep scores were also correlated with reduced odds of preterm delivery (OR = 0.733, *p* = 0.047). For the IUI-D cohort, elevated sleep scores showed a positive correlative association with miscarriage odds (OR = 1.976, *p* = 0.041). Notably, mild anxiety was observed in 97% of IVF/ICSI-D participants, and rising anxiety scores were positively correlated with higher odds of live birth (OR = 1.185, *p* = 0.043) and cesarean delivery (OR = 1.192, *p* = 0.029).

**Conclusion:**

Sleep quality displays correlative predictive links to pregnancy outcomes among donor insemination recipients, with distinct correlative patterns observed across different ART modalities. Mild anxiety exhibits a positive correlative association with favourable live birth rates within the IVF/ICSI-D subgroup, alongside a correlative link to higher cesarean delivery prevalence. Our observational associations support the clinical value of routine sleep screening and individualized psychological support for this patient cohort.

## 1 Introduction

The World Health Organization (WHO) defines infertility as a disease of the reproductive system. It is diagnosed when a clinical pregnancy fails to occur after 12 months or more of regular, unprotected intercourse. Current estimates indicate that approximately one in six individuals of reproductive age worldwide will experience infertility at some point. The condition results from dysfunction in the reproductive systems of one or both partners. Male factors contribute to 30–50% of all cases (1). Donor sperm-assisted reproductive technology (ART) provides a crucial treatment option for couples facing male-factor infertility. Intrauterine insemination with donor sperm (IUI-D) has become the standard treatment for severe male-factor infertility, such as azoospermia. In contrast, *in vitro* fertilization/intracytoplasmic sperm injection with donor sperm (IVF/ICSI-D) is reserved for patients who have not achieved pregnancy after IUI-D cycles or who present with concurrent female-factor pathology, such as tubal obstruction or severe endometriosis. The clinical use of donor sperm-assisted reproduction has expanded substantially in recent years. Registry data from the UK Human Fertilization and Embryology Authority (HFEA) clearly document this trend. Between 2006 and 2016, IUI-D cycles increased by 40%, while IVF/ICSI-D cycles rose by 377% (2).

Patients undergoing donor insemination face unique psychological stressors. Traditional cultural expectations regarding biological paternity create substantial pressure. The anonymous nature of donor sperm introduces additional uncertainty into family dynamics. Treatment costs impose a significant financial burden. Together, these factors increase the risk of anxiety, depression, and sleep disturbances in this population. Empirical evidence supports these clinical observations. One study reported mean Self-Rating Anxiety Scale (SAS) scores of 43.98 ± 6.80 and Self-Rating Depression Scale (SDS) scores of 46.03 ± 10.61 in this group. These values substantially exceed national norms (29.78 ± 10.46 and 41.88 ± 10.57, respectively) (3). Sleep disruption is another prevalent concern. Population-based surveys indicate that 14–40% of the general public report sleep disorders, with prevalence rising markedly among ART population, reaching 46 to 57% (4).

Previous research has primarily focused on the safety of pregnancy outcomes in donor insemination-assisted reproduction (5–8). However, the relationship between patient health status and treatment success remains poorly understood, particularly regarding physical and psychological health indicators. Sleep quality, anxiety symptoms, depressive tendencies, and somatic complaints may correlate with reproductive outcomes. Their specific effects on clinical pregnancy, live birth, and preterm delivery have not been clearly established. To address this gap, the present study utilized prospective cohort data from the Reproductive Center of West China Second University Hospital, Sichuan University, to quantity associations between these health factors and pregnancy outcomes in the donor insemination population. Potential confounding variables, including age, body mass index (BMI), and educational attainment, were also evaluated. The primary objectives were to determine whether psychological and somatic factors correlate with key pregnancy outcomes and to examine whether these associations differ between intrauterine insemination with IUI-D and IVF/ICSI-D. The findings aim to inform clinical practice by providing a foundation for integrated interventions combining psychological counseling, sleep hygiene education, and cognitive behavioral therapy for insomnia (CBT-I).

## 2 Materials and methods

### 2.1 Setting and participants

A total of 758 consecutive patients who underwent donor insemination at the outpatient clinic were enrolled between June 2020 and January 2024. Eligible participants included those receiving IUI-D (donor-sperm intrauterine insemination), IVF/ICSI-D (donor-sperm IVF/ICSI).

Three exclusion criteria were applied. Patients were excluded if their treatment cycle began more than 3 months after initial registration. Those who canceled their cycle for personal reasons were also excluded. For patients undergoing IVF/ICSI cycles, exclusion also applied if no embryo transfer was performed during the current cycle and the subsequent frozen–thawed embryo transfer was scheduled more than 3 months later. Of the initially enrolled 516 IUI-D patients, 4 participants were excluded from the final analytical dataset due to incomplete pregnancy outcome follow-up data. The final analytical sample for IUI-D was therefore 512 cycles, while the IVF/ICSI-D analytical sample remained 234 after excluding 8 patients without embryo transfer.

### 2.2 Psychological assessment tools

Four internationally validated scales were employed for the standardized assessment of somatic symptoms, anxiety, depression, and sleep quality. The Patient Health Questionnaire-15 (PHQ-15) (9) was used to evaluate somatic symptom burden. The Generalized Anxiety Disorder-7 (GAD-7) (10) assessed anxiety severity. The Patient Health Questionnaire-9 (PHQ-9) (11) measured depressive symptoms. The Pittsburgh Sleep Quality Index (PSQI) (12) quantified sleep quality. All instruments utilized Likert-scale scoring, with higher scores indicating greater symptom severity.

All four scales have confirmed reliability and validity among Chines infertile populations in previous cohort studies (13, 14). The self-administered questionnaire design avoids blood collection and instrumental examinations, which reduces patients’ outpatient burden and is highly suitable for large-sample prospective data collection, consistent with mainstream research methods in reproductive medicine.

A standardized severity classification was applied across all four scales (15). Scores of 0–4 indicated minimal symptom burden, 5–9 mild burden, 10–14 moderate burden, and 15 or higher severe burden. Scale reliability was confirmed prior to data collection, with Cronbach’s alpha coefficients exceeding 0.70 for all four instruments, indicating acceptable internal consistency. All assessments were conducted at the time of patient registration.

### 2.3 Data collection

Data was collected from the hospital’s electronic medical records. Demographic variables included age, ethnicity, residential area, educational attainment, and BMI. Infertility-related variables comprised infertility type and reproductive history.

The primary outcome was the clinical pregnancy rate, defined as the proportion of cycles resulting in a clinical pregnancy. Clinical pregnancy was confirmed by ultrasonographic visualization of an intrauterine gestational sac 35 days after embryo transfer.

$$\begin{array}{l} \text{Clinical pregnancy rate}\\ =\frac{\text{Number of cycles with clinical pregnany}}{\text{Total}\;\text{embryo transfer cycles}}\times 100\% \end{array}$$

Secondary outcomes included the live birth rate, preterm birth rate (gestational-age-based endpoint), low birth weight rate (birth-weight-based endpoint), multiple pregnancy rate, and incidence of pregnancy complications. The live birth rate was defined as the proportion of cycles resulting in a live birth. The preterm birth rate was defined as the proportion of clinically pregnant cycles resulting in preterm delivery. The low birth weight rate was defined as the proportion of live births with a neonatal weight below 2,500 grams.

$$\text{Live birth rate}=\frac{\text{Number of live birth cycles}}{\text{Total embryo transfer cycles}}\times 100\%$$

$$\text{Preterm birth rate}=\frac{\text{Number of cycles with preterm birth}}{\text{Total clinically pregnant cycles}}\times 100\%$$

$$\mathrm{Low}\;\text{birth weight rate}=\frac{\text{Number of}\;\mathrm{low}\;\text{birth weight infants}}{\text{Total live births cases}}\times 100\%$$

$$\begin{array}{l} \text{Multiple pregnancy rate}\\ =\frac{\text{Number of multiple pregnancy cycles}}{\text{Total clinically pregnant cycles}}\times 100\% \end{array}$$

$$\begin{array}{l} \text{Incidence of pregnancy complication}\\ =\frac{\text{Number of cycles with pregnancy complications}}{\text{Total clinically pregnant cycles}}\times 100\% \end{array}$$

Preterm birth was defined as delivery before 37 completed weeks of gestation. Low birth weight was defined as a neonatal birth weight below 2,500 grams. Preterm birth (based on gestational age) and low birth weight (based on neonatal weight) are separate outcomes and were analysed as distinct endpoints.

All above pregnancy endpoints were calculated uniformly using the formulas listed above, and descriptive statistics are presented in Table 1 of the Results section.

**Table 1:** Pregnancy outcomes of patients receiving donor sperm assisted reproduction.

Pregnancy outcomes	IVF/ICSI-D	IUI-D
Pregnancy rate	60.7 (142/234)	16.3 (84/512)
Live birth rate	54.3 (127/234)	15.4 (79/512)
Cesarean delivery rate	74.4 (97/127)	59.5 (47/79)
Premature birth rate	15.0 (19/127)	13.9 (11/79)
Multiple birth rate	12.6 (16/142)	4.8 (4/84)
Low birth weight rate	11.8 (15/127)	0 (0/79)
Incidence of complications	19.4 (24/124)	9.5 (8/84)

(1) Preterm birth and low birth weight represent two separate study endpoints, defined by gestational age and neonatal birth weight, respectively.

(2) A total of 516 IUI-D patients were initially enrolled; 4 cases were excluded from statistical analyses owing to missing pregnancy outcome data, leading to a final analytical sample of 512 IUI-D cycles. The IVF/ICSI-D cohort enrolled 242 patients initially, with 8 excluded for no embryo transfer, resulting in 234 valid cycles for analysis.

### 2.4 Statistical analysis

Statistical analyses were performed using SPSS version 27.0 (IBM Corp., Armonk, NY, USA). Normality tests indicated that age, BMI, and infertility duration were not normally distributed. Therefore, group comparisons for these variables were conducted using the Mann–Whitney U test. Categorical variables, including educational attainment and employment status, were analyzed using the chi-square test. Categorical data are presented as frequencies and percentages.

Candidate confounding variables were determined based on systematic literature review and clinical reproductive experience, covering three categories:

1. Demographic covariates: age, ethnicity, residential area, remarriage status, occupation, educational attainment, BMI;
2. Reproductive covariates: infertility duration, infertility type, previous reproductive history;
3. Exposure indicators: total scores of anxieties, depression, somatic symptoms and sleep quality.

All clinically meaningful reproductive confounders were retained in the variable pool without arbitrary exclusion.

Before constructing multivariable logistic regression models, multicollinearity diagnosis was performed by calculating variance inflation factor (VIF) for all independent variables. The VIF values of all variables included in the regression models were less than 5, demonstrating no severe multicollinearity among psychological scales and demographic covariates. Full VIF results are provided in Supplementary Table S1.

#### 2.4.1 Analysis workflow

Univariate logistic regression was first performed for all candidate variables. All independent variables with univariate *p* < 0.1, and variables with clear clinical significance regardless of *p*-values, were incorporated into fully adjusted multivariable logistic regression models to adequately control confounding bias. Sensitivity analyses were further carried out by removing educational attainment from covariates to test the robustness of core associations.

Statistical significance was defined as a two-tailed *p*-value less than 0.05.

### 2.5 Ethical considerations

This study was approved by the Medical Ethics Committee of West China Second University Hospital, Sichuan University (approval number: 2024248). The requirement for written informed consent was waived because the study protocol did not involve the collection of identifiable personal information. Specifically, patient names, identification numbers, telephone numbers, and biometric identifiers were not recorded.

## 3 Results

### 3.1 Characteristics of participants

A total of 758 patients were enrolled. Of these, 242 underwent IVF/ICSI-D. Eight cases were excluded due to non-transfer, resulting in 234 valid cases. The mean age was 30.07 ± 4.40 years, and the mean duration of infertility was 4.13 ± 3.25 years. Among the 516 patients who received IUI-D, the mean age was 28.79 ± 3.69 years, and the mean duration of infertility was 3.43 ± 2.39 years.

Primary infertility accounted for over 70% of all cases. A history of previous reproductive issues was reported in 73.97% of the IVF/ICSI-D cohort, compared with only 12.71% of the IUI-D cohort. Most patients resided in urban areas (74.8 and 83.0%, respectively; see Table 2). Employment was reported in over 80% of cases (86.8 and 91.9%, respectively; Table 2). More than 50% of participants had attained a college education or higher (54.3 and 69.5%, respectively; see Table 2). Normal body weight was observed in over 65% of cases (67.9 and 70.9%, respectively). Detailed data are presented in Table 2.

**Table 2:** Baseline characteristics of patients by treatment modality and parity status.

Indicator	IVF/ICSI-D (142/234)			IUI-D (84/516)		
	Nullipara	Multipara	Total	Nullipara	Multipara	Total
Age	31.0 ± 5.0	29.6 ± 4.0	30.1 ± 4.4	28.8 ± 3.7	28.6 ± 3.5	28.8 ± 3.7
Infertility duration	4.8 ± 4.1	3.7 ± 2.5	4.1 ± 3.3	3.4 ± 2.5	3.6 ± 2.1	3.4 ± 2.4
Remarriage (female)	13 (5.6)	12 (5.1)	25 (10.7)	7 (1.4)	30 (5.8)	37 (7.2)
Ethnicity						
Han	87 (37.2)	137 (58.5)	224 (95.7)	82 (15.9)	414 (80.2)	496 (96.1)
Other ethnic minorities	5 (2.1)	5 (2.1)	10 (4.3)	2 (0.4)	18 (3.5)	20 (3.9)
Infertility type						
Primary [*n*%]	65 (27.8)	110 (47.0)	175 (74.8)	315 (61.1)	58 (11.2)	373 (72.3)
Secondary [*n*%]	27 (11.5)	32 (13.7)	59 (25.2)	117 (22.7)	26 (5.0)	143 (27.7)
Reproductive history						
With [*n*%]	63 (26.9)	109 (46.6)	172 (73.5)	95 (18.4)	17 (3.3)	112 (21.7)
Without [*n*%]	29 (12.4)	33 (14.1)	62 (26.5)	337 (65.3)	67 (13.0)	404 (78.3)
Residential area						
Urban [*n*%]	72 (30.8)	103 (44.0)	175 (74.8)	358 (69.4)	70 (13.6)	428 (83.0)
Rural [*n*%]	20 (8.5)	39 (16.7)	59 (25.2)	74 (14.3)	14 (2.7)	88 (17.0)
Occupation						
Non-employed [*n*%]	8 (3.4)	23 (9.8)	31 (13.2)	37 (7.2)	5 (1.0)	42 (8.2)
Employed [*n*%]	84 (35.9)	119 (50.9)	203 (86.8)	395 (76.6)	79 (15.3)	474 (91.9)
Educational attainment						
Junior High School and Below [*n*%]	27 (11.5)	34 (14.5)	61 (26.1)	63 (12.2)	20 (3.9)	83 (16.1)
High School/Secondary Technical School [*n*%]	17 (7.3)	29 (12.4)	46 (19.7)	63 (12.2)	11 (2.1)	74 (14.4)
College and above [*n*%]	48 (20.5)	79 (33.8)	127 (54.3)	306 (59.3)	53 (10.2)	359 (69.5)
BMI						
BMI < 18.5 [*n*%]	9 (3.8)	16 (6.8)	25 (10.7)	49 (9.5)	6 (1.2)	55 (10.7)
18.5 ≤ BMI < 24 [*n*%]	60 (25.6)	99 (42.3)	159 (67.9)	303 (58.7)	63 (21.2)	366 (70.9)

### 3.2 Psychological and somatic health factors

**Figure 1:**
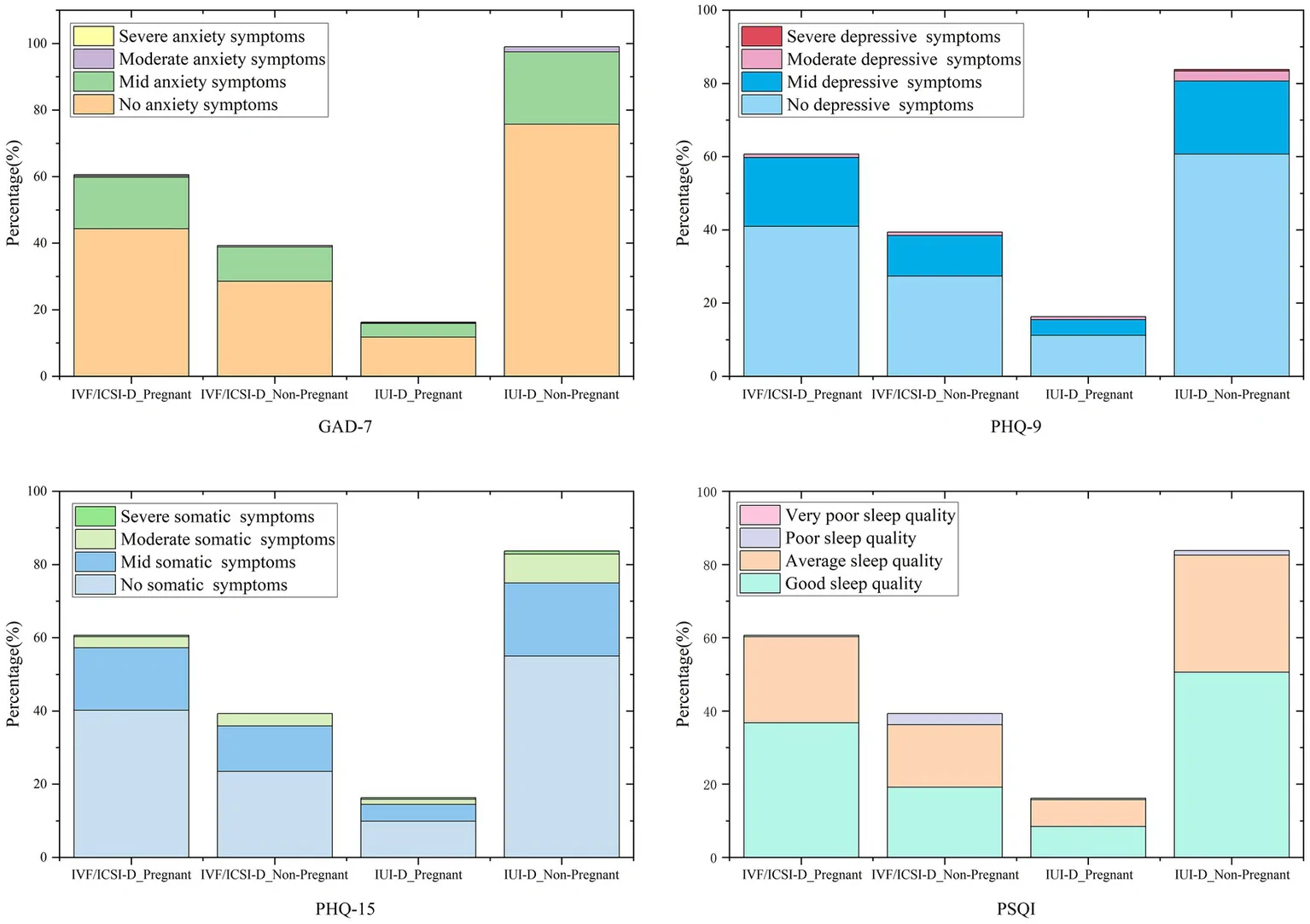
Psychological and somatic health parameters by treatment modality and pregnancy status.

Health status varied according to treatment modality, as shown in Figure 1. The highest proportions of asymptomatic patients and those with normal sleep quality were observed in the non-pregnant IUI-D subgroup. Mild anxiety, depression, and somatic symptoms were more prevalent in the IVF/ICSI-D cohort. Severe symptoms accounted for less than 3% of cases in both cohorts, indicating minimal serious morbidity.

Pregnancy status showed divergent associations across modalities. Among IVF/ICSI-D patients, the pregnant subgroup exhibited more favorable health profiles, characterized by lower anxiety severity, reduced depressive symptoms, fewer somatic complaints, and better sleep quality compared to their non-pregnant counterparts. Conversely, in IUI-D patients, the pregnant subgroup demonstrated poorer health status, with higher anxiety severity, more pronounced depressive symptoms, a greater burden of somatic complaints, and worse sleep quality.

### 3.3 Pregnancy outcomes

We separately calculated all predefined pregnancy outcome indicators for IVF/ICSI-D and IUI-D cohorts and summarized descriptive data in Table 1, without conducting inter-group comparative statistical analysis.

Pregnancy outcomes included clinical pregnancy status, live birth, cesarean delivery, preterm birth, low birth weight, twin pregnancy, and pregnancy-related complications, with preterm birth and low birth weight treated as independent analytical indicators, as detailed in Table 1.

Complications included hypertensive disorders of pregnancy, preeclampsia or eclampsia, gestational diabetes mellitus, placenta previa, placental abruption, premature rupture of membranes, postpartum hemorrhage, oligohydramnios or polyhydramnios, intrahepatic cholestasis, cholelithiasis, uterine fibroids, thrombocytopenia, fetal distress, and antepartum hemorrhage.

The clinical pregnancy rate of patients receiving IUI-D was 16.3%, with a live birth rate of 15.4%. The above descriptive data only reflects subgroup distribution characteristics. We further constructed fully adjusted logistic regression models to explore independent predictors of pregnancy outcomes, as shown in Table 3.

**Table 3:** Multivariable logistic regression analyses of psychological and sleep indicators associated with pregnancy outcomes among infertile women: overall population and conception-mode stratified subgroups.

Stratified population	Independent variable	Clinical pregnancy OR (95%CI), *p*	Live birth OR (95%CI), *p*	Cesarean Section OR (95%CI), *p*	Preterm birth OR (95%CI), *p*	Multiple pregnancy OR (95%CI), *p*
Overall infertile population	Anxiety total score	1.091 (0.998–1.192), 0.056	1.140 (1.039–1.250), **0.006**	1.127 (1.018–1.247), **0.021**	0.970 (0.767–1.227), 0.801	1.070 (0.836–1.370), 0.589
	Depression total score	0.912 (0.831–1.001), 0.052	0.888 (0.806–0.978), **0.016**	0.910 (0.816–1.013), 0.086	1.001 (0.792–1.266), 0.992	1.017 (0.786–1.315), 0.900
	Sleep total score	1.010 (0.924–1.104), 0.834	0.992 (0.905–1.088), 0.865	1.021 (0.921–1.131), 0.699	0.755 (0.595–0.957), **0.020**	0.935 (0.719–1.215), 0.613
	Somatic symptoms total score	1.016 (0.952–1.083), 0.636	1.018 (0.953–1.088), 0.601	0.963 (0.891–1.041), 0.345	1.095 (0.939–1.278), 0.248	0.972 (0.800–1.181), 0.775
IUI-D subgroup	Anxiety total score	1.053 (0.913–1.190), 0.381	1.070 (0.934–1.226), 0.326	0.929 (0.825–1.192), 0.929	0.886 (0.601–1.305), 0.539	1.233 (0.543–2.798), 0.616
	Depression total score	0.887 (0.769–1.023), 0.099	0.884 (0.763–1.024), 0.099	0.935 (0.772–1.132), 0.489	1.001 (0.691–1.450), 0.996	0.327 (0.102–1.047), 0.060
	Sleep total score	1.145 (0.999–1.313), 0.051	1.089 (0.947–1.253), 0.232	1.085 (0.912–1.291), 0.355	0.767 (0.519–1.133), 0.183	0.579 (0.272–1.231), 0.156
	Somatic symptoms total score	1.025 (0.930–1.130), 0.618	1.026 (0.929–1.134), 0.610	0.970 (0.849–1.108), 0.649	1.169 (0.899–1.519), 0.244	2.010 (1.070–3.775), **0.030**
IVF/ICSI-D subgroup	Anxiety total score	1.091 (0.932–1.279), 0.279	1.185 (1.005–1.397), **0.043**	1.193 (1.019–1.396), **0.029**	0.953 (0.693–1.310), 0.766	0.982 (0.716–1.348), 0.912
	Depression total score	1.037 (0.888–1.211), 0.643	0.971 (0.832–1.134), 0.714	0.975 (0.838–1.133), 0.737	1.071 (0.789–1.453), 0.659	1.235 (0.924–1.650), 0.154
	Sleep total score	0.827 (0.712–0.960), **0.013**	0.851 (0.734–0.985), **0.031**	0.916 (0.792–1.059), 0.235	0.732 (0.539–0.995), **0.046**	0.967 (0.736–1.272), 0.812
	Somatic symptoms total score	0.965 (0.865–1.077), 0.528	0.976 (0.876–1.087), 0.654	0.930 (0.834–1.037), 0.193	1.036 (0.851–1.262), 0.722	0.860 (0.676–1.093), 0.218

Model adjustment: all models are adjusted for confounding factors including age, body mass index (BMI), infertility duration, and three-category education level (with junior high school and below as the reference group). Statistical definitions: OR, odds ratio; 95%CI, 95% confidence interval; *p*-values < 0.05 indicate statistically significant differences and are presented in bold. Outcome description: no statistically significant associations were found between psychological and sleep indicators and the two outcomes of pregnancy complications and low birth weight (a weight-based endpoint independent of gestational-age-derived preterm birth) in the three groups. The complete full-variable regression data is available in Supplementary Table S2.

### 3.4 Predictors of pregnancy outcomes

Based on the baseline and descriptive pregnancy data above, we built fully adjusted logistic regression models taking psychological and sleep indicators as exposures and all pregnancy endpoints as dependent variables. Analyses were implemented for the overall population, IUI-D subgroup and IVF/ICSI-D subgroup respectively, with core results summarized in Table 3.

Binary logistic regression was performed using age, infertility duration, BMI and three-category education as adjustment confounders. Dependent variables covered clinical pregnancy, live birth, cesarean delivery, preterm delivery, multiple gestation, low birth weight and pregnancy complications.

### 3.5 Sensitivity analysis

To verify whether core associations were interfered by educational attainment, we excluded education level from covariates and re-established all regression models for the overall population, IUI-D and IVF/ICSI-D subgroups, with comparison results displayed in Table 4.

**Table 4:** Sensitivity analyses of multivariable logistic regression for associations between psychological and sleep indicators and pregnancy outcomes: overall population and conception-mode stratified subgroups.

Variable	Outcome	Overall population main model OR (95%CI), *p*	Overall population sensitivity model OR (95%CI), *p*	IUI-D main model OR (95%CI), *p*	IUI-D sensitivity model OR (95%CI), *p*	IVF/ICSI-D main model OR (95%CI), *p*	IVF/ICSI-D sensitivity model OR (95%CI), *p*
Anxiety total score	Live birth	1.140 (1.039–1.250), 0.006	1.135 (1.035–1.245), 0.007	1.070 (0.934–1.226), 0.326	1.074 (0.936–1.232), 0.309	1.185 (1.005–1.397), 0.043	1.209 (1.027–1.423), 0.022
Anxiety total score	Cesarean section	1.127 (1.018–1.247), 0.021	1.124 (1.016–1.244), 0.024	0.929 (0.825–1.192), 0.929	0.990 (0.881–1.112), 0.912	1.193 (1.019–1.396), 0.029	1.196 (1.026–1.394), 0.022
Depression total score	Live birth	0.888 (0.806–0.978), 0.016	0.886 (0.805–0.976), 0.014	0.884 (0.763–1.024), 0.099	0.886 (0.765–1.027), 0.107	0.971 (0.832–1.134), 0.714	0.963 (0.825–1.124), 0.630
Sleep total score	Clinical pregnancy	1.010 (0.924–1.104), 0.834	1.011 (0.926–1.104), 0.808	1.145 (0.999–1.313), 0.051	1.135 (0.992–1.300), 0.066	0.827 (0.712–0.960), 0.013	0.829 (0.715–0.961), 0.013
Sleep total score	Live birth	0.992 (0.905–1.088), 0.865	0.994 (0.907–1.090), 0.830	1.089 (0.947–1.253), 0.232	1.083 (0.942–1.246), 0.262	0.851 (0.734–0.985), 0.031	0.845 (0.730–0.978), 0.024
Sleep total score	Preterm birth	0.755 (0.595–0.957), 0.020	0.759 (0.598–0.963), 0.023	0.767 (0.519–1.133), 0.183	0.768 (0.520–1.135), 0.187	0.732 (0.539–0.995), 0.046	0.725 (0.533–0.987), 0.041
Somatic symptoms total score	Multiple pregnancy	0.972 (0.800–1.181), 0.775	0.972 (0.800–1.181), 0.775	2.010 (1.070–3.775), 0.030	2.010 (1.070–3.775), 0.030	0.860 (0.676–1.093), 0.218	0.860 (0.676–1.093), 0.218

Sensitivity analyses were conducted by excluding educational attainment from covariates to evaluate the robustness of associations derived from fully adjusted primary multivariable logistic regression models.

After omitting educational level from the adjusted variables, all statistically significant associations between psychological and sleep indicators and pregnancy outcomes observed in the main models remained consistent. The magnitudes of odds ratios, 95% confidence intervals and *p* values only exhibited minor fluctuations, with no qualitative shift in statistical significance.

These findings indicated that the core associations were not substantially confounded by educational attainment, confirming the robustness of our primary results (Table 4).

Table 4 only summarizes core statistically significant results. Full pairwise comparison data of main and sensitivity models across all subgroups are sorted into Supplementary Table S3.

## 4 Discussion

This study examined 758 patients undergoing donor sperm-assisted reproduction at a single tertiary referral center. The analysis systematically evaluated the associations between sleep quality, psychological factors, and somatic symptoms with pregnancy outcomes. Method-specific differences were identified across the IVF/ICSI-D and IUI-D pathways. These findings provide empirical evidence to optimize the clinical management of this patient population.

### 4.1 Impact of sleep quality on pregnancy outcomes

Statistical models revealed significant correlative links between sleep scores and pregnancy endpoints in this cohort. Across the full sample and IVF/ICSI-D subgroup, poorer sleep was correlatively associated with higher preterm birth prevalence, indicating consistent observational correlation between sleep disturbance and preterm delivery across donor insemination patients. The direction and magnitude of these associations varied according to the treatment modality. These findings address a gap in existing literature concerning sleep and reproductive outcomes in patients undergoing donor insemination.

In the IVF/ICSI-D cohort, sleep quality demonstrated independent associations with clinical pregnancy, live birth, and preterm birth (all *p* < 0.05). Each one-unit increase in PSQI sleep score was associated with 17.3% lower odds of clinical pregnancy (OR 0.827), 15.0% lower odds of live birth (OR 0.850), and 26.7% lower odds of preterm delivery (OR 0.733). Several hypothetical biological correlative pathways may explain these statistical links. Observational data shows sleep irregularity correlates with disrupted HPO axis balance and abnormal gonadotropin secretion, which aligns with poorer follicle development and reduced oocyte quality in clinical cohorts. Previous studies have demonstrated that poor sleep quality is associated with 15% fewer retrieved oocytes and 18% fewer mature oocytes (16, 17). Hemodynamic alterations constitute a second mechanism. Poor sleep correlates with higher norepinephrine concentrations in previous studies, a biomarker that shows correlative links to weaker uterine blood flow, diminished endometrial receptivity and lower embryo implantation likelihood in observational research (18, 19).

Activation of inflammatory pathways may underlie the association between sleep quality and preterm birth. Shortened sleep duration correlates with elevated inflammatory markers, a biomarker profile associated with unstable intrauterine environments in published cohort data (20). Additionally, nocturnal intermittent hypoxia resulting from sleep-disordered breathing may impair placental function and compromise fetal oxygenation and nutrient delivery (21–24). Epidemiological evidence indicates that a mid-sleep time of ≤ 02:45 is associated with a 64% higher risk of preterm birth (OR 1.64) (25), and poor subjective sleep quality correlates with an increased risk of late preterm birth (relative risk 1.19) (26). Collectively, these findings suggest that sleep timing, circadian rhythm, and sleep quality significantly influence pregnancy outcomes.

Divergent correlative patterns between sleep and miscarriage risk across subgroups may correspond to differences in ovarian stimulation regimens used for IUI-D and IVF/ICSI-D. IVF relies on controlled ovarian hyperstimulation, whereby exogenous gonadotropins sustain relatively steady systemic hormonal profiles. By contrast, IUI employs natural ovulation cycles or mild ovarian stimulation, yielding a delicate luteal endocrine state. In the IUI-D cohort, poorer sleep correlates with greater hormonal fluctuation and higher rates of corpus luteum dysfunction, which aligns with elevated miscarriage odds in adjusted regression models (OR = 1.976, *p* = 0.041).

In the IUI-D cohort, sleep quality was significantly associated with miscarriage risk (OR 1.976, *p* = 0.041). The association with clinical pregnancy approached statistical significance (OR 1.145, *p* = 0.051). These divergent effects likely reflect differences in treatment protocols. IUI-D relies on natural or mildly stimulated ovulation, with requires greater endocrine stability. Luteal phase insufficiency and hormonal fluctuations resulting from sleep disruption may impair early embryonic development, elevating miscarriage risk. In contrast, IVF/ICSI-D involves controlled ovarian hyperstimulation and *in vitro* fertilization. However, post-transfer implantation and development remain dependent on intrauterine homeostasis. Thus, sleep quality thus correlates with multiple stages: conception establishment, pregnancy maintenance, and preterm birth prevention.

### 4.2 Anxiety and pregnancy outcomes: bidirectional associations

Anxiety demonstrated modality-specific associations within this cohort, Anxiety displayed heterogeneous correlative associations across the two ART subgroups, rather than causal effects on reproductive endpoints. In the IVF/ICSI-D group, anxiety scores were positively correlated with live birth (OR 1.185, *p* = 0.043) and cesarean delivery (OR 1.192, *p* = 0.029). These divergent correlative patterns contradict the common assumption that anxiety maintains universally negative correlative links with all pregnancy endpoints. Further research is needed to elucidate the underlying mechanisms.

The IVF/ICSI-D group exhibited a 97% prevalence of mild anxiety. In contrast, Swedish nationwide registry studies included populations with moderate-to-severe anxiety requiring medical diagnosis (27). This difference in severity may explain the observed heterogeneity. A cohort study conducted in Egypt further validated that mild anxiety does not impair IVF pregnancy outcomes, with adverse reductions in conception rates only observed among patients with moderate or severe anxiety (28). Mild anxiety correlates with more consistent self-care behaviours, a behavioural pattern that may partially explain the observed correlative association with favourable live birth outcomes. Patients with mild anxiety tend to follow prenatal protocols more closely and report clinical symptoms timely; such behavioural patterns show correlative links to timely risk screening and higher live birth prevalence in this cohort (29, 30). This interpretation aligns with evidence that moderate psychological stress can activate coping resources and optimize health-related decision-making (30). Nevertheless, this positive correlation could be affected by two methodological limitations, namely sample selection and timing bias. In terms of selection bias, patients with mild anxiety tend to sustain better treatment compliance and present lower loss-to-follow-up rates (31). Regarding measurement timing, anxiety assessed at baseline only captures transient anticipatory stress prior to treatment rather than chronic pathological anxiety (32).

Notably, the positive association between mild anxiety and live birth should be interpreted as hypothesis-generating rather than causal. It may be affected by residual confounding, selection bias and non-linear effects, and cannot be generalized to patients with moderate-to-severe anxiety.

The positive association between anxiety and cesarean delivery is consistent with findings from multiple studies (33–35). This relationship operates through several pathways. Psychologically, Anxiety symptoms show correlative links to heightened perceived labour pain and increased preference for cesarean delivery among participants. Patients may perceive cesarean delivery as a way to reduce delivery risks, particularly among younger women with lower educational attainment (35). Physiologically, Anxiety correlates with sympathetic arousal and higher serum cortisol, biomarkers that align with weaker uterine contraction and longer labour duration in observational obstetric studies (33, 34). Additionally, placental effects may also contribute, as anxiety-related hormonal changes can affect fetal growth and indirectly prompt decisions for surgical delivery (33, 34).

Clinical interaction patterns further mediate this association. Patients with elevated anxiety scores tend to voice more clinical worries, a pattern correlatively linked to clinicians’ higher perceived obstetric risk in routine care. Combined with defensive medical practices, this leads to an increased rate of cesarean recommendations. Concurrently, anxious patients tend to rely more heavily on physician advice, facilitating convergence in decision-making toward surgical delivery (33, 35). Socio-cultural factors, including perceptions that cesarean deliveries are faster and safer, as well as disparities in healthcare access, reinforce this tendency (33, 35).

### 4.3 Other predictors of pregnancy outcomes

Beyond sleep quality and anxiety, several additional predictors were identified. In the IVF/ICSI-D cohort, Advanced maternal age showed a negative correlative association with clinical pregnancy rates (OR 0.933, *p* = 0.035), matching widely documented age-correlated fertility patterns across ART cohorts (6, 8). Notably, Elevated somatic symptom burden displayed a positive correlative association with multiple pregnancy prevalence within the IUI-D subgroup (OR = 2.010, *p* = 0.030). Chronic somatic complaints and inflammatory states correlate with asynchronous follicle maturation, a physiological pattern associated with higher odds of multiple ovulation in prior research. For the overall population, sleep showed a marginal protective trend against low birth weight (OR = 0.751, *p* = 0.052), implying long-term sleep disturbance may restrict intrauterine fetal growth and require prenatal monitoring attention. Both Oocyte quantity and quality decrease after age 35, reducing implantation potential. BMI was independently associated with complications (OR 1.222, *p* = 0.005). Obesity shows consistent correlative links to higher gestational diabetes incidence, with insulin resistance proposed as one intermediate correlative biomarker and inflammatory pathways (24). In the IUI-D cohort, somatic symptom scores correlated positively with multiple gestation (OR 1.922, *p* = 0.042). Endocrine dysfunction and inflammatory states underlying somatic symptoms may affect follicular development, thereby increasing the probability of multiple pregnancies. This association requires validation in larger samples.

Baseline characteristics differed significantly between cohorts. The IVF/ICSI-D group was older (30.1 ± 4.4 years versus 28.8 ± 3.7 years), had a longer duration of infertility (4.1 ± 3.3 years versus 3.4 ± 2.4 years), and was more likely to have a previous reproductive history (73.5% versus 21.7%). These differences likely reflect indication-based treatment selection. IVF/ICSI-D is typically reserved for patients with failed IUI-D or concurrent female pathology, who present with more complex fertility profiles. This contextual factor contributes to the observed heterogeneity in predictors across treatment modalities.

### 4.4 Clinical implications

Sensitivity analyses excluding educational attainment verified all core heterogeneous associations remained stable without obvious changes in OR and *p* values, which fully proves the reliability of our regression results.

Consistent with the divergent predictive effects observed overall, IVF/ICSI-D and IUI-D cohorts (Tables 3, 4), we propose stratified, quantifiable intervention strategies for clinical practice:

1. IVF/ICSI-D population: routine sleep assessment is clinically recommended for donor insemination patients; sleep-support interventions show correlative links to reduced preterm birth rates in published ART cohorts. Standard reproductive counselling is suitable for mildly anxious patients; timely psychological support for those with moderate–severe anxiety correlates with fewer non-medically indicated cesarean deliveries in observational data.
2. IUI-D population: mandatory sleep assessment at enrollment, with targeted sleep optimization to decrease early miscarriage incidence.
3. Quantitative clinical threshold: patients with PSQI ≥7 immediately receive sleep hygiene guidance and CBT-I, and standardized regular follow-up schedules are formulated for mildly anxious patients.

## 5 Limitations and future directions

This study has several limitations. First, the single-center design may limit generalizability of the findings, which require validation in multi-center studies with larger sample sizes. Second, all sleep and mental assessments rely solely on self-reported questionnaires without objective indicators such as polysomnography, dynamic cortisol and reproductive sex hormones, inevitably introducing recall and subjective scoring bias. Third, sperm quality and embryo grading were not included, potentially leaving residual confounding unaddressed. Fourth, this single-center observational design cannot support definitive causal conclusions about correlative shifts in pregnancy outcomes following clinical interventions. Fifth, although variance inflation factor values were below the conventional threshold for severe multicollinearity, residual collinearity among psychological-sleep related scales might still bias our regression-based effect estimates. Future studies are encouraged to adopt larger samples or structural equation modelling to better disentangle the independent effects of these interrelated constructs.

Subsequent research should prioritize multi-center randomized trials, incorporate objective sleep monitoring, adopt advanced analytical strategies including structural equation modelling, include additional clinical variables, and investigate individualized interventions alongside genetic regulatory mechanisms.

## Data Availability

The raw data supporting the conclusions of this article will be made available by the authors, without undue reservation.
