# Supplementary material for: Heterogeneous effects of sleep and psychological health on pregnancy outcomes in donor insemination: a prospective cohort study
Source: Front Public Health. 2026 Aug 26;14:1821081. doi: 10.3389/fpubh.2026.1821081 (PMC13553487; doi:10.3389/fpubh.2026.1821081)
Supplement: Supplementary file 1 [file Data_Sheet_1.PDF]

Appendix

Table S1

Tolerance and variance inflation factor (VIF) of exposure variables

Variables	Tolerance	Variance Inflation Factor (VIF)
Total anxiety score	0.396	2.524
Total depression score	0.353	2.831
Total sleep quality score	0.614	1.63
Total somatic symptom score	0.488	2.049

Table S2: part A

Full Results of Multivariable Logistic Regression Analysis for Overall Infertile Population: Associations Between Psychological and Sleep Indicators and Pregnancy Outcomes

Pregnancy Outcome	Covariate	β	OR	95% CI	P-value
	Age	0.031	1.032	0.985–1.080	0.183
	Body Mass Index (BMI)	-0.029	0.972	0.920–1.026	0.299
	Anxiety Total Score	0.087	1.091	0.998–1.192	0.056
	Depression Total Score	-0.092	0.912	0.831–1.001	0.052
Clinical Pregnancy	Sleep Total Score	0.01	1.01	0.924–1.104	0.834
	Somatic Symptoms Total Score	0.016	1.016	0.952–1.083	0.636
	Education Level 2	-0.498	0.608	0.404–0.914	0.017
	Education Level 3	-1.59	0.204	0.073–0.569	0.002
	Infertility Duration	-0.04	0.961	0.900–1.027	0.239

Pregnancy Outcome	Covariate	β	OR	95% CI	P-value
Live Birth	Age	0.019	1.019	0.972–1.069	0.433
	BMI	-0.047	0.954	0.902–1.010	0.106
	Anxiety Total Score	0.131	1.14	1.039–1.250	0.006
	Depression Total Score	-0.119	0.888	0.806–0.978	0.016
	Sleep Total Score	-0.008	0.992	0.905–1.088	0.865
	Somatic Symptoms Total Score	0.018	1.018	0.953–1.088	0.601
	Education Level 2	-0.294	0.746	0.487–1.140	0.176
	Education Level 3	-1.259	0.284	0.101–0.799	0.017
	Infertility Duration	-0.012	0.988	0.923–1.057	0.718
Cesarean Section	Age	0.045	1.046	0.992–1.102	0.095
	BMI	-0.007	0.993	0.933–1.057	0.827
	Anxiety Total Score	0.119	1.127	1.018–1.247	0.021
	Depression Total Score	-0.095	0.91	0.816–1.013	0.086
	Sleep Total Score	0.02	1.021	0.921–1.131	0.699
	Somatic Symptoms Total Score	-0.037	0.963	0.891–1.041	0.345
	Education Level 2	-0.302	0.739	0.463–1.182	0.207
	Education Level 3	-1.424	0.241	0.068–0.856	0.028
	Infertility Duration	0.006	1.006	0.936–1.082	0.865
Preterm Birth	Age	0.051	1.052	0.946–1.171	0.347
	BMI	-0.01	0.99	0.872–1.123	0.871
	Anxiety Total Score	-0.03	0.97	0.767–1.227	0.801
	Depression Total Score	0.001	1.001	0.792–1.266	0.992
	Sleep Total Score	-0.281	0.755	0.595–0.957	0.02

Pregnancy Outcome	Covariate	β	OR	95% CI	P-value
	Somatic Symptoms Total Score	0.091	1.095	0.939–1.278	0.248
	Education Level 2	0.326	1.385	0.493–3.887	0.536
	Education Level 3	-17.969	0	0.000–.	0.998
	Infertility Duration	0.044	1.045	0.915–1.192	0.518
	Age	0.049	1.05	0.951–1.158	0.334
	BMI	0.107	1.113	0.999–1.240	0.053
Pregnancy Complications	Anxiety Total Score	0.132	1.141	0.954–1.364	0.149
	Depression Total Score	-0.209	0.812	0.653–1.009	0.061
	Sleep Total Score	0.047	1.048	0.863–1.274	0.636
	Somatic Symptoms Total Score	0.031	1.032	0.889–1.198	0.679
	Education Level 2	-0.006	0.994	0.401–2.463	0.989
	Education Level 3	-17.991	0	0.000–.	0.998
	Infertility Duration	0.036	1.037	0.911–1.179	0.582
	Age	0.07	1.073	0.944–1.218	0.282
	BMI	0.043	1.044	0.899–1.213	0.572
	Anxiety Total Score	0.068	1.07	0.836–1.370	0.589
	Depression Total Score	0.017	1.017	0.786–1.315	0.9
Multiple Pregnancy	Sleep Total Score	-0.068	0.935	0.719–1.215	0.613
	Somatic Symptoms Total Score	-0.029	0.972	0.800–1.181	0.775
	Education Level 2	-0.342	0.71	0.246–2.046	0.526
	Education Level 3	-17.822	0	0.000–.	0.998
	Infertility Duration	0.125	1.133	0.980–1.309	0.092
Low Birth Weight	Age	0.062	1.063	0.937–1.207	0.341

Pregnancy Outcome	Covariate	β	OR	95% CI	P-value
	BMI	0.048	1.049	0.909–1.211	0.509
	Anxiety Total Score	0.052	1.054	0.797–1.394	0.713
	Depression Total Score	0.044	1.045	0.786–1.390	0.76
	Sleep Total Score	-0.287	0.751	0.563–1.002	0.052
	Somatic Symptoms Total Score	-0.029	0.971	0.791–1.193	0.781
	Education Level 2	0.105	1.11	0.341–3.615	0.862
	Education Level 3	-17.727	0	0.000–.	0.998
	Infertility Duration	0.049	1.05	0.903–1.220	0.526

Table S2: part B

Full Results of Multivariable Logistic Regression Analysis for IUI-D Subgroup: Associations Between Psychological and Sleep Indicators and Pregnancy Outcomes

Pregnancy Outcome	Covariate	β	OR	95%CI	P-value
Clinical Pregnancy	Age	-0.041	0.96	0.878–1.049	0.353
	Body Mass Index (BMI)	-0.044	0.957	0.869–1.054	0.379
	Anxiety Total Score	0.052	1.053	0.913–1.190	0.381
	Depression Total Score	-0.119	0.887	0.769–1.023	0.099
	Sleep Total Score	0.135	1.145	0.999–1.313	0.051
	Somatic Symptoms Total Score	0.025	1.025	0.930–1.130	0.618
	Education Level 2	-0.519	0.595	0.370–0.956	0.032
	Education Level 3	-1.573	0.207	0.072–0.594	0.003
	Infertility Duration	-0.061	0.941	0.849–1.043	0.239

Pregnancy Outcome	Covariate	β	OR	95%CI	P-value
Live Birth	Age	-0.039	0.962	0.882–1.047	0.369
	BMI	-0.072	0.931	0.845–1.026	0.145
	Anxiety Total Score	0.067	1.07	0.934–1.226	0.326
	Depression Total Score	-0.124	0.884	0.763–1.024	0.099
	Sleep Total Score	0.085	1.089	0.947–1.253	0.232
	Somatic Symptoms Total Score	0.026	1.026	0.929–1.134	0.61
	Education Level 2	-0.311	0.733	0.465–1.155	0.181
	Education Level 3	-1.271	0.28	0.099–0.792	0.017
	Infertility Duration	-0.01	0.99	0.893–1.098	0.837
Cesarean Section	Age	0.005	1.005	0.925–1.090	0.907
	BMI	-0.031	0.969	0.881–1.065	0.504
	Anxiety Total Score	-0.074	0.929	0.825–1.192	0.929
	Depression Total Score	-0.067	0.935	0.772–1.132	0.489
	Sleep Total Score	0.082	1.085	0.912–1.291	0.355
	Somatic Symptoms Total Score	-0.03	0.97	0.849–1.108	0.649
	Education Level 2	-0.31	0.733	0.451–1.190	0.209
	Education Level 3	-1.419	0.242	0.068–0.861	0.029
	Infertility Duration	-0.015	0.985	0.888–1.091	0.787
Preterm Birth	Age	0.013	1.013	0.874–1.175	0.874
	BMI	0.025	1.025	0.874–1.201	0.748
	Anxiety Total Score	-0.121	0.886	0.601–1.305	0.539
	Depression Total Score	0.001	1.001	0.691–1.450	0.996

Pregnancy Outcome	Covariate	β	OR	95%CI	P-value
	Sleep Total Score	-0.265	0.767	0.519–1.133	0.183
	Somatic Symptoms Total Score	0.156	1.169	0.899–1.519	0.244
	Education Level 2	0.318	1.374	0.489–3.862	0.541
	Education Level 3	-17.952	0	0.000–.	0.998
	Infertility Duration	0.043	1.044	0.910–1.194	0.52
	Age	-0.006	0.994	0.871–1.133	0.926
	BMI	0.198	1.219	1.057–1.404	0.007
	Anxiety Total Score	0.178	1.195	0.976–1.464	0.082
Pregnancy Complications	Depression Total Score	-0.157	0.854	0.669–1.091	0.201
	Sleep Total Score	-0.045	0.956	0.758–1.206	0.704
	Somatic Symptoms Total Score	0.006	1.006	0.839–1.206	0.944
	Education Level 2	-0.167	0.846	0.213–3.360	0.817
	Education Level 3	-18.642	0	0.000–.	0.998
	Infertility Duration	0.057	1.059	0.905–1.237	0.471
	Age	-0.02	0.98	0.830–1.155	0.801
	BMI	0.073	1.076	0.904–1.280	0.405
	Anxiety Total Score	0.21	1.233	0.543–2.798	0.616
	Depression Total Score	-1.119	0.327	0.102–1.047	0.06
Multiple Pregnancy	Sleep Total Score	-0.547	0.579	0.272–1.231	0.156
	Somatic Symptoms Total Score	0.7	2.01	1.070–3.775	0.03
	Education Level 2	-0.131	0.877	0.144–5.340	0.984
	Education Level 3	-17.987	0	0.000–.	0.998
	Infertility Duration	0.148	1.16	0.962–1.399	0.119

Pregnancy Outcome	Covariate	β	OR	95%CI	P-value
Low Birth Weight	Age	0.061	1.063	0.936–1.206	0.342
	BMI	0.047	1.048	0.908–1.210	0.51
	Anxiety Total Score	0.051	1.052	0.796–1.393	0.714
	Depression Total Score	0.043	1.044	0.785–1.389	0.761
	Sleep Total Score	-0.286	0.751	0.563–1.002	0.052
	Somatic Symptoms Total Score	-0.029	0.971	0.791–1.193	0.781
	Education Level 2	0.104	1.11	0.341–3.614	0.862
	Education Level 3	-17.726	0	0.000–.	0.998
	Infertility Duration	0.048	1.05	0.903–1.220	0.527

Table S2: Part C
Full Multivariable Logistic Regression Results for IVF/ICSI-D Subgroup

Pregnancy Outcome	Covariate	β	OR	95% CI	P-value
Clinical Pregnancy	Age	-0.043	0.958	0.887–1.034	0.27
	Body Mass Index (BMI)	-0.043	0.958	0.874–1.051	0.368
	Anxiety Total Score	0.087	1.091	0.932–1.279	0.279
	Depression Total Score	0.037	1.037	0.888–1.211	0.643
	Sleep Total Score	-0.19	0.827	0.712–0.960	0.013
	Somatic Symptoms Total Score	-0.035	0.965	0.865–1.077	0.528
	Education Level 2	0.115	1.122	0.570–2.210	0.739
	Education Level 3	-0.12	0.887	0.123–6.385	0.905
	Infertility Duration	-0.062	0.94	0.847–1.042	0.238

Pregnancy Outcome	Covariate	β	OR	95% CI	P-value
Live Birth	Age	-0.041	0.96	0.890–1.036	0.295
	BMI	-0.073	0.93	0.848–1.020	0.122
	Anxiety Total Score	0.17	1.185	1.005–1.397	0.043
	Depression Total Score	-0.029	0.971	0.832–1.134	0.714
	Sleep Total Score	-0.162	0.851	0.734–0.985	0.031
	Somatic Symptoms Total Score	-0.025	0.976	0.876–1.087	0.654
	Education Level 2	0.351	1.421	0.723–2.792	0.308
	Education Level 3	-0.475	0.635	0.227–11.386	0.635
	Infertility Duration	-0.011	0.989	0.892–1.097	0.836
Cesarean Section	Age	0.006	1.006	0.933–1.086	0.871
	BMI	-0.032	0.968	0.884–1.061	0.491
	Anxiety Total Score	0.176	1.193	1.019–1.396	0.029
	Depression Total Score	-0.026	0.975	0.838–1.133	0.737
	Sleep Total Score	-0.088	0.916	0.792–1.059	0.235
	Somatic Symptoms Total Score	-0.072	0.93	0.834–1.037	0.193
	Education Level 2	0.035	1.036	0.529–2.026	0.919
	Education Level 3	-0.176	0.839	0.118–5.943	0.86
	Infertility Duration	-0.014	0.986	0.890–1.092	0.788
Preterm Birth	Age	0.014	1.014	0.879–1.171	0.844
	BMI	0.026	1.026	0.877–1.201	0.745
	Anxiety Total Score	-0.048	0.953	0.693–1.310	0.766
	Depression Total Score	0.069	1.071	0.789–1.453	0.659
	Sleep Total Score	-0.312	0.732	0.539–0.995	0.046

Pregnancy Outcome	Covariate	β	OR	95% CI	P-value
	Somatic Symptoms Total Score	0.036	1.036	0.851–1.262	0.722
	Education Level 2	0.862	2.368	0.573–9.787	0.234
	Education Level 3	-18.278	0	0.000–.	1
	Infertility Duration	0.047	1.048	0.883–1.243	0.592
	Age	-0.007	0.993	0.876–1.127	0.916
	BMI	0.2	1.221	1.060–1.407	0.006
	Anxiety Total Score	0.187	1.206	0.984–1.477	0.071
	Depression Total Score	-0.142	0.867	0.681–1.105	0.249
Pregnancy Complications	Sleep Total Score	-0.046	0.955	0.757–1.205	0.701
	Somatic Symptoms Total Score	0.007	1.007	0.840–1.207	0.942
	Education Level 2	-0.013	0.987	0.343–2.839	0.98
	Education Level 3	-18.657	0	0.000–.	1
	Infertility Duration	0.058	1.059	0.906–1.238	0.469
	Age	-0.021	0.98	0.832–1.154	0.806
	BMI	0.074	1.077	0.905–1.282	0.403
	Anxiety Total Score	-0.018	0.982	0.716–1.348	0.912
	Depression Total Score	0.211	1.235	0.924–1.650	0.154
Multiple Pregnancy	Sleep Total Score	-0.033	0.967	0.736–1.272	0.812
	Somatic Symptoms Total Score	-0.151	0.86	0.676–1.093	0.218
	Education Level 2	0.166	1.181	0.328–4.249	0.799
	Education Level 3	-17.994	0	0.000–.	1
	Infertility Duration	0.149	1.161	0.963–1.400	0.118
Low Birth Weight	Age	0.062	1.063	0.937–1.207	0.341

Pregnancy Outcome	Covariate	β	OR	95% CI	P-value
	BMI	0.048	1.049	0.909–1.211	0.509
	Anxiety Total Score	0.052	1.054	0.797–1.394	0.713
	Depression Total Score	0.044	1.045	0.786–1.390	0.76
	Sleep Total Score	-0.287	0.751	0.563–1.002	0.052
	Somatic Symptoms Total Score	-0.029	0.971	0.791–1.193	0.781
	Education Level 2	0.105	1.11	0.341–3.615	0.862
	Education Level 3	-17.727	0	0.000–.	0.998
	Infertility Duration	0.049	1.05	0.903–1.220	0.526

Table Notes

Model adjustment: All models were adjusted for age, body mass index (BMI), infertility duration, and three-category education level (reference group: junior high school and below; Education Level 2 = high school/technical secondary school, Education Level 3 = college and above).

Statistical definitions: β Coefficient = Logistic regression coefficient; Odds Ratio (OR) = Exp(β); 95%CI = 95% confidence interval; P-values < 0.05 indicate statistically significant differences and are presented in bold

Table S3: part A

Main Models and Sensitivity Analyses for the Overall Infertile Population

Pregnancy Outcome	Covariate	Main Model β Coefficient	Main Model OR (95%CI)	Main Model P-value	Sensitivity Model β Coefficient	Sensitivity Model OR (95%CI)	Sensitivity Model P-value
Clinical	Age	0.031	1.032 (0.985–1.080)	0.183	0.01	1.010 (0.966–1.055)	0.674
Pregnancy	BMI	-0.029	0.972 (0.920–1.026)	0.299	-0.016	0.984 (0.933–1.038)	0.546

Pregnancy Outcome	Covariate	Main Model β Coefficient	Main Model OR (95%CI)	Main Model P-value	Sensitivity Model β Coefficient	Sensitivity Model OR (95%CI)	Sensitivity Model P-value
	Anxiety total score	0.087	1.091 (0.998–1.192)	0.056	0.081	1.081 (0.992–1.184)	0.075
	Depression total score	-0.092	0.912 (0.831–1.001)	0.052	-0.093	0.911 (0.831–1.000)	0.048
	Sleep total score	0.01	1.010 (0.924–1.104)	0.834	0.011	1.011 (0.926–1.104)	0.808
	Somatic symptoms total score	0.016	1.016 (0.952–1.083)	0.636	0.025	1.025 (0.961–1.092)	0.452
	Education level 2	-0.498	0.608 (0.404–0.914)	0.017	—	—	—
	Education level 3	-1.59	0.204 (0.073–0.569)	0.002	—	—	—
	Infertility duration	-0.04	0.961 (0.900–1.027)	0.239	-0.014	0.986 (0.924–1.052)	0.671
	Age	0.019	1.019 (0.972–1.069)	0.433	0.002	1.002 (0.957–1.049)	0.939
	BMI	-0.047	0.954 (0.902–1.010)	0.106	-0.038	0.963 (0.911–1.018)	0.188
Live Birth	Anxiety total score	0.131	1.140 (1.039–1.250)	0.006	0.127	1.135 (1.035–1.245)	0.007
	Depression total score	-0.119	0.888 (0.806–0.978)	0.016	-0.121	0.886 (0.805–0.976)	0.014
	Sleep total score	-0.008	0.992 (0.905–1.088)	0.865	-0.006	0.994 (0.907–1.090)	0.83
	Somatic symptoms total score	0.018	1.018 (0.953–1.088)	0.601	0.025	1.025 (0.960–1.094)	0.459
	Education level 2	-0.294	0.746 (0.487–1.140)	0.176	—	—	—
	Education level 3	-1.259	0.284 (0.101–0.799)	0.017	—	—	—
	Infertility duration	-0.012	0.988 (0.923–1.057)	0.718	0.007	1.007 (0.943–1.076)	0.832
	Age	0.045	1.046 (0.992–1.102)	0.095	0.03	1.031 (0.979–1.085)	0.251
	BMI	-0.007	0.993 (0.933–1.057)	0.827	0.002	1.002 (0.942–1.066)	0.947
Cesarean Section	Anxiety total score	0.119	1.127 (1.018–1.247)	0.021	0.117	1.124 (1.016–1.244)	0.024
	Depression total score	-0.095	0.910 (0.816–1.013)	0.086	-0.097	0.907 (0.814–1.011)	0.078
	Sleep total score	0.02	1.021 (0.921–1.131)	0.699	0.024	1.024 (0.924–1.135)	0.652

Pregnancy Outcome	Covariate	Main Model β Coefficient	Main Model OR (95%CI)	Main Model P-value	Sensitivity Model β Coefficient	Sensitivity Model OR (95%CI)	Sensitivity Model P-value
	Somatic symptoms total score	-0.037	0.963 (0.891–1.041)	0.345	-0.031	0.970 (0.898–1.047)	0.435
	Education level 2	-0.302	0.739 (0.463–1.182)	0.207	—	—	—
	Education level 3	-1.424	0.241 (0.068–0.856)	0.028	—	—	—
	Infertility duration	0.006	1.006 (0.936–1.082)	0.865	0.025	1.025 (0.954–1.101)	0.497
	Age	0.051	1.052 (0.946–1.171)	0.347	0.043	1.044 (0.941–1.157)	0.417
	BMI	-0.01	0.990 (0.872–1.123)	0.871	-0.004	0.996 (0.878–1.130)	0.952
	Anxiety total score	-0.03	0.970 (0.767–1.227)	0.801	-0.029	0.972 (0.770–1.226)	0.808
	Depression total score	0.001	1.001 (0.792–1.266)	0.992	0.004	1.004 (0.797–1.265)	0.973
Preterm Birth	Sleep total score	-0.281	0.755 (0.595–0.957)	0.02	-0.276	0.759 (0.598–0.963)	0.023
	Somatic symptoms total score	0.091	1.095 (0.939–1.278)	0.248	0.095	1.099 (0.943–1.281)	0.226
	Education level 2	0.326	1.385 (0.493–3.887)	0.536	—	—	—
	Education level 3	-17.969	0.000 (0.000–.)	0.998	—	—	—
	Infertility duration	0.044	1.045 (0.915–1.192)	0.518	0.052	1.053 (0.927–1.196)	0.427
	Age	0.049	1.050 (0.951–1.158)	0.334	-0.006	0.994 (0.871–1.133)	0.926
	BMI	0.107	1.113 (0.999–1.240)	0.053	0.198	1.219 (1.057–1.404)	0.007
	Anxiety total score	0.132	1.141 (0.954–1.364)	0.149	0.178	1.195 (0.976–1.464)	0.082
	Depression total score	-0.209	0.812 (0.653–1.009)	0.061	-0.157	0.854 (0.669–1.091)	0.201
Pregnancy Complications	Sleep total score	0.047	1.048 (0.863–1.274)	0.636	-0.045	0.956 (0.758–1.206)	0.704
	Somatic symptoms total score	0.031	1.032 (0.889–1.198)	0.679	0.006	1.006 (0.839–1.206)	0.944
	Education level 2	-0.006	0.994 (0.401–2.463)	0.989	—	—	—
	Education level 3	-17.991	0.000 (0.000–.)	0.998	—	—	—

Pregnancy Outcome	Covariate	Main Model β Coefficient	Main Model OR (95%CI)	Main Model P-value	Sensitivity Model β Coefficient	Sensitivity Model OR (95%CI)	Sensitivity Model P-value
Multiple Pregnancy	Infertility duration	0.036	1.037 (0.911–1.179)	0.582	0.057	1.059 (0.905–1.237)	0.471
	Age	0.07	1.073 (0.944–1.218)	0.282	-0.02	0.980 (0.830–1.155)	0.801
	BMI	0.043	1.044 (0.899–1.213)	0.572	0.073	1.076 (0.904–1.280)	0.405
	Anxiety total score	0.068	1.070 (0.836–1.370)	0.589	0.21	1.233 (0.543–2.798)	0.616
	Depression total score	0.017	1.017 (0.786–1.315)	0.9	-1.119	0.327 (0.102–1.047)	0.06
	Sleep total score	-0.068	0.935 (0.719–1.215)	0.613	-0.547	0.579 (0.272–1.231)	0.156
	Somatic symptoms total score	-0.029	0.972 (0.800–1.181)	0.775	0.7	2.010 (1.070–3.775)	0.03
	Education level 2	-0.342	0.710 (0.246–2.046)	0.526	—	—	—
	Education level 3	-17.822	0.000 (0.000–.)	0.998	—	—	—
Low Birth Weight	Infertility duration	0.125	1.133 (0.980–1.309)	0.092	0.148	1.160 (0.962–1.399)	0.119
	Age	0.062	1.063 (0.937–1.207)	0.341	0.061	1.063 (0.936–1.206)	0.342
	BMI	0.048	1.049 (0.909–1.211)	0.509	0.047	1.048 (0.908–1.210)	0.51
	Anxiety total score	0.052	1.054 (0.797–1.394)	0.713	0.051	1.052 (0.796–1.393)	0.714
	Depression total score	0.044	1.045 (0.786–1.390)	0.76	0.043	1.044 (0.785–1.389)	0.761
	Sleep total score	-0.287	0.751 (0.563–1.002)	0.052	-0.286	0.751 (0.563–1.002)	0.052
	Somatic symptoms total score	-0.029	0.971 (0.791–1.193)	0.781	-0.029	0.971 (0.791–1.193)	0.781
	Education level 2	0.105	1.110 (0.341–3.615)	0.862	—	—	—
	Education level 3	-17.727	0.000 (0.000–.)	0.998	—	—	—
	Infertility duration	0.049	1.050 (0.903–1.220)	0.526	0.048	1.050 (0.903–1.220)	0.527

Table S3: part B

Main Models and Sensitivity Analyses for IUI-D Subgroup

Pregnancy Outcome	Covariate	Main Model β Coefficient	Main Model OR (95%CI)	Main Model P-value	Sensitivity Model β Coefficient	Sensitivity Model OR (95%CI)	Sensitivity Model P-value
Clinical Pregnancy	Age	-0.041	0.960 (0.878–1.049)	0.353	-0.016	0.984 (0.918–1.056)	0.659
	BMI	-0.044	0.957 (0.869–1.054)	0.379	-0.011	0.989 (0.913–1.071)	0.782
	Anxiety total score	0.052	1.053 (0.913–1.190)	0.381	0.044	1.045 (0.914–1.195)	0.515
	Depression total score	-0.119	0.887 (0.769–1.023)	0.099	-0.118	0.889 (0.770–1.028)	0.108
	Sleep total score	0.135	1.145 (0.999–1.313)	0.051	0.127	1.135 (0.992–1.300)	0.066
	Somatic symptoms total score	0.025	1.025 (0.930–1.130)	0.618	0.03	1.030 (0.935–1.135)	0.547
	Education level 2	-0.519	0.595 (0.370–0.956)	0.032	—	—	—
	Education level 3	-1.573	0.207 (0.072–0.594)	0.003	—	—	—
	Infertility duration	-0.061	0.941 (0.849–1.043)	0.239	0.024	1.024 (0.922–1.138)	0.653
Live Birth	Age	-0.039	0.962 (0.882–1.047)	0.369	-0.035	0.966 (0.899–1.038)	0.342
	BMI	-0.072	0.931 (0.845–1.026)	0.145	-0.026	0.974 (0.897–1.059)	0.54
	Anxiety total score	0.067	1.070 (0.934–1.226)	0.326	0.071	1.074 (0.936–1.232)	0.309
	Depression total score	-0.124	0.884 (0.763–1.024)	0.099	-0.121	0.886 (0.765–1.027)	0.107
	Sleep total score	0.085	1.089 (0.947–1.253)	0.232	0.08	1.083 (0.942–1.246)	0.262
	Somatic symptoms total score	0.026	1.026 (0.929–1.134)	0.61	0.03	1.030 (0.933–1.137)	0.56
	Education level 2	-0.311	0.733 (0.465–1.155)	0.181	—	—	—
	Education level 3	-1.271	0.280 (0.099–0.792)	0.017	—	—	—
	Infertility duration	-0.01	0.990 (0.893–1.098)	0.837	0.036	1.037 (0.931–1.155)	0.43
Cesarean Section	Age	0.005	1.005 (0.925–1.090)	0.907	-0.017	0.983 (0.900–1.074)	0.71
	BMI	-0.031	0.969 (0.881–1.065)	0.504	0.035	1.035 (0.939–1.141)	0.484
	Anxiety total score	-0.074	0.929 (0.825–1.192)	0.929	-0.011	0.990 (0.881–1.112)	0.912
	Depression total score	-0.067	0.935 (0.772–1.132)	0.489	-0.067	0.936 (0.773–1.134)	0.497

Pregnancy Outcome	Covariate	Main Model β Coefficient	Main Model OR (95%CI)	Main Model P-value	Sensitivity Model β Coefficient	Sensitivity Model OR (95%CI)	Sensitivity Model P-value
	Sleep total score	0.082	1.085 (0.912–1.291)	0.355	0.083	1.087 (0.914–1.293)	0.348
	Somatic symptoms total score	-0.03	0.970 (0.849–1.108)	0.649	-0.026	0.974 (0.852–1.113)	0.702
	Education level 2	-0.31	0.733 (0.451–1.190)	0.209	—	—	—
	Education level 3	-1.419	0.242 (0.068–0.861)	0.029	—	—	—
	Infertility duration	-0.015	0.985 (0.888–1.091)	0.787	0.071	1.074 (0.945–1.219)	0.274
	Age	0.013	1.013 (0.874–1.175)	0.874	0.008	1.008 (0.841–1.208)	0.932
	BMI	0.025	1.025 (0.874–1.201)	0.748	-0.058	0.943 (0.757–1.176)	0.628
Preterm Birth	Anxiety total score	-0.121	0.886 (0.601–1.305)	0.539	-0.127	0.881 (0.598–1.297)	0.52
	Depression total score	0.001	1.001 (0.691–1.450)	0.996	0.015	1.015 (0.699–1.473)	0.938
	Sleep total score	-0.265	0.767 (0.519–1.133)	0.183	-0.264	0.768 (0.520–1.135)	0.187
	Somatic symptoms total score	0.156	1.169 (0.899–1.519)	0.244	0.161	1.174 (0.902–1.526)	0.236
	Education level 2	0.318	1.374 (0.493–3.862)	0.541	—	—	—
	Education level 3	-17.952	0.000 (0.000–.)	0.998	—	—	—
	Infertility duration	0.043	1.044 (0.910–1.194)	0.52	0.067	1.069 (0.835–1.369)	0.598
	Age	-0.006	0.994 (0.871–1.133)	0.926	-0.006	0.994 (0.871–1.133)	0.926
	BMI	0.198	1.219 (1.057–1.404)	0.007	0.198	1.219 (1.057–1.404)	0.007
	Anxiety total score	0.178	1.195 (0.976–1.464)	0.082	0.178	1.195 (0.976–1.464)	0.082
Pregnancy Complications	Depression total score	-0.157	0.854 (0.669–1.091)	0.201	-0.157	0.854 (0.669–1.091)	0.201
	Sleep total score	-0.045	0.956 (0.758–1.206)	0.704	-0.045	0.956 (0.758–1.206)	0.704
	Somatic symptoms total score	0.006	1.006 (0.839–1.206)	0.944	0.006	1.006 (0.839–1.206)	0.944
	Education level 2	-0.167	0.846 (0.213–3.360)	0.817	—	—	—

Pregnancy Outcome	Covariate	Main Model β Coefficient	Main Model OR (95%CI)	Main Model P-value	Sensitivity Model β Coefficient	Sensitivity Model OR (95%CI)	Sensitivity Model P-value
Multiple Pregnancy	Education level 3	-18.642	0.000 (0.000–.)	0.998	—	—	—
	Infertility duration	0.057	1.059 (0.905–1.237)	0.471	0.057	1.059 (0.905–1.237)	0.471
	Age	-0.02	0.980 (0.830–1.155)	0.801	-0.02	0.980 (0.830–1.155)	0.801
	BMI	0.073	1.076 (0.904–1.280)	0.405	0.073	1.076 (0.904–1.280)	0.405
	Anxiety total score	0.21	1.233 (0.543–2.798)	0.616	0.21	1.233 (0.543–2.798)	0.616
	Depression total score	-1.119	0.327 (0.102–1.047)	0.06	-1.119	0.327 (0.102–1.047)	0.06
	Sleep total score	-0.547	0.579 (0.272–1.231)	0.156	-0.547	0.579 (0.272–1.231)	0.156
	Somatic symptoms total score	0.7	2.010 (1.070–3.775)	0.03	0.7	2.010 (1.070–3.775)	0.03
	Education level 2	-0.131	0.877 (0.144–5.340)	0.984	—	—	—
	Education level 3	-17.987	0.000 (0.000–.)	0.998	—	—	—
Low Birth Weight	Infertility duration	0.148	1.160 (0.962–1.399)	0.119	0.148	1.160 (0.962–1.399)	0.119
	Age	0.061	1.063 (0.936–1.206)	0.342	0.061	1.063 (0.936–1.206)	0.342
	BMI	0.047	1.048 (0.908–1.210)	0.51	0.047	1.048 (0.908–1.210)	0.51
	Anxiety total score	0.051	1.052 (0.796–1.393)	0.714	0.051	1.052 (0.796–1.393)	0.714
	Depression total score	0.043	1.044 (0.785–1.389)	0.761	0.043	1.044 (0.785–1.389)	0.761
	Sleep total score	-0.286	0.751 (0.563–1.002)	0.052	-0.286	0.751 (0.563–1.002)	0.052
	Somatic symptoms total score	-0.029	0.971 (0.791–1.193)	0.781	-0.029	0.971 (0.791–1.193)	0.781
	Education level 2	0.104	1.110 (0.341–3.614)	0.862	—	—	—
	Education level 3	-17.726	0.000 (0.000–.)	0.998	—	—	—
	Infertility duration	0.048	1.050 (0.903–1.220)	0.527	0.048	1.050 (0.903–1.220)	0.527

Table S3: part C

Main Models and Sensitivity Analyses for IVF/ICSI-D Conception Subgroup

Pregnancy Outcome	Covariate	Main Model β Coefficient	Main Model OR (95%CI)	Main Model P-value	Sensitivity Model β Coefficient	Sensitivity Model OR (95%CI)	Sensitivity Model P-value
Clinical Pregnancy	Age	-0.043	0.958 (0.887–1.034)	0.27	-0.043	0.958 (0.889–1.033)	0.264
	BMI	-0.043	0.958 (0.874–1.051)	0.368	-0.044	0.957 (0.873–1.049)	0.368
	Anxiety total score	0.087	1.091 (0.932–1.279)	0.279	0.093	1.098 (0.940–1.283)	0.24
	Depression total score	0.037	1.037 (0.888–1.211)	0.643	0.035	1.036 (0.887–1.210)	0.654
	Sleep total score	-0.19	0.827 (0.712–0.960)	0.013	-0.188	0.829 (0.715–0.961)	0.013
	Somatic symptoms total score	-0.035	0.965 (0.865–1.077)	0.528	-0.039	0.962 (0.863–1.072)	0.476
	Education level 2	0.115	1.122 (0.570–2.210)	0.739	—	—	—
	Education level 3	-0.12	0.887 (0.123–6.385)	0.905	—	—	—
	Infertility duration	-0.062	0.940 (0.847–1.042)	0.238	-0.062	0.940 (0.851–1.038)	0.218
Live Birth	Age	-0.041	0.960 (0.890–1.036)	0.295	-0.033	0.967 (0.899–1.041)	0.376
	BMI	-0.073	0.930 (0.848–1.020)	0.122	-0.078	0.925 (0.844–1.013)	0.094
	Anxiety total score	0.17	1.185 (1.005–1.397)	0.043	0.19	1.209 (1.027–1.423)	0.022
	Depression total score	-0.029	0.971 (0.832–1.134)	0.714	-0.038	0.963 (0.825–1.124)	0.63
	Sleep total score	-0.162	0.851 (0.734–0.985)	0.031	-0.168	0.845 (0.730–0.978)	0.024
	Somatic symptoms total score	-0.025	0.976 (0.876–1.087)	0.654	-0.027	0.974 (0.876–1.071)	0.62
	Education level 2	0.351	1.421 (0.723–2.792)	0.308	—	—	—
	Education level 3	-0.475	0.635 (0.227–11.386)	0.635	—	—	—
	Infertility duration	-0.011	0.989 (0.892–1.097)	0.836	-0.024	0.976 (0.885–1.077)	0.627
Cesarean Section	Age	0.006	1.006 (0.933–1.086)	0.871	0.007	1.007 (0.936–1.084)	0.843
	BMI	-0.032	0.968 (0.884–1.061)	0.491	-0.033	0.968 (0.884–1.060)	0.479

Pregnancy Outcome	Covariate	Main Model β Coefficient	Main Model OR (95%CI)	Main Model P-value	Sensitivity Model β Coefficient	Sensitivity Model OR (95%CI)	Sensitivity Model P-value
	Anxiety total score	0.176	1.193 (1.019–1.396)	0.029	0.179	1.196 (1.026–1.394)	0.022
	Depression total score	-0.026	0.975 (0.838–1.133)	0.737	-0.023	0.977 (0.841–1.135)	0.764
	Sleep total score	-0.088	0.916 (0.792–1.059)	0.235	-0.079	0.924 (0.801–1.067)	0.348
	Somatic symptoms total score	-0.072	0.930 (0.834–1.037)	0.193	-0.081	0.922 (0.828–1.027)	0.138
	Education level 2	0.035	1.036 (0.529–2.026)	0.919	—	—	—
	Education level 3	-0.176	0.839 (0.118–5.943)	0.86	—	—	—
	Infertility duration	-0.014	0.986 (0.890–1.092)	0.788	-0.009	0.991 (0.899–1.092)	0.852
	Age	0.014	1.014 (0.879–1.171)	0.844	0.02	1.020 (0.896–1.161)	0.762
	BMI	0.026	1.026 (0.877–1.201)	0.745	0.019	1.019 (0.868–1.196)	0.819
	Anxiety total score	-0.048	0.953 (0.693–1.310)	0.766	-0.001	0.999 (0.749–1.333)	0.996
	Depression total score	0.069	1.071 (0.789–1.453)	0.659	0.052	1.053 (0.792–1.399)	0.722
	Sleep total score	-0.312	0.732 (0.539–0.995)	0.046	-0.321	0.725 (0.533–0.987)	0.041
Preterm Birth	Somatic symptoms total score	0.036	1.036 (0.851–1.262)	0.722	0.02	1.020 (0.841–1.238)	0.84
	Education level 2	0.862	2.368 (0.573–9.787)	0.234	—	—	—
	Education level 3	-18.278	0.000 (0.000–.)	1	—	—	—
	Infertility duration	0.047	1.048 (0.883–1.243)	0.592	0.033	1.033 (0.887–1.203)	0.673
	Age	-0.007	0.993 (0.876–1.127)	0.916	-0.007	0.993 (0.876–1.127)	0.916
	BMI	0.2	1.221 (1.060–1.407)	0.006	0.2	1.221 (1.060–1.407)	0.006
Pregnancy Complications	Anxiety total score	0.187	1.206 (0.984–1.477)	0.071	0.187	1.206 (0.984–1.477)	0.071
	Depression total score	-0.142	0.867 (0.681–1.105)	0.249	-0.142	0.867 (0.681–1.105)	0.249
	Sleep total score	-0.046	0.955 (0.757–1.205)	0.701	-0.046	0.955 (0.757–1.205)	0.701

Pregnancy Outcome	Covariate	Main Model β Coefficient	Main Model OR (95%CI)	Main Model P-value	Sensitivity Model β Coefficient	Sensitivity Model OR (95%CI)	Sensitivity Model P-value
	Somatic symptoms total score	0.007	1.007 (0.840–1.207)	0.942	0.007	1.007 (0.840–1.207)	0.942
	Education level 2	-0.013	0.987 (0.343–2.839)	0.98	—	—	—
	Education level 3	-18.657	0.000 (0.000–.)	1	—	—	—
Multiple Pregnancy	Infertility duration	0.058	1.059 (0.906–1.238)	0.469	0.058	1.059 (0.906–1.238)	0.469
	Age	-0.021	0.980 (0.832–1.154)	0.806	-0.021	0.979 (0.836–1.147)	0.795
	BMI	0.074	1.077 (0.905–1.282)	0.403	-0.078	0.925 (0.844–1.013)	0.094
	Anxiety total score	-0.018	0.982 (0.716–1.348)	0.912	0.188	1.207 (0.551–2.648)	0.616
	Depression total score	0.211	1.235 (0.924–1.650)	0.154	-0.939	0.391 (0.129–1.189)	0.098
	Sleep total score	-0.033	0.967 (0.736–1.272)	0.812	-0.534	0.586 (0.268–1.284)	0.182
	Somatic symptoms total score	-0.151	0.860 (0.676–1.093)	0.218	0.559	1.749 (1.005–3.041)	0.048
	Education level 2	0.166	1.181 (0.328–4.249)	0.799	—	—	—
	Education level 3	-17.994	0.000 (0.000–.)	1	—	—	—
	Infertility duration	0.149	1.161 (0.963–1.400)	0.118	0.165	1.180 (0.801–1.737)	0.402
	Age	0.062	1.063 (0.937–1.207)	0.341	0.062	1.063 (0.937–1.207)	0.341
	BMI	0.048	1.049 (0.909–1.211)	0.509	0.048	1.049 (0.909–1.211)	0.509
	Anxiety total score	0.052	1.054 (0.797–1.394)	0.713	0.052	1.054 (0.797–1.394)	0.713
Low Birth Weight	Depression total score	0.044	1.045 (0.786–1.390)	0.76	0.044	1.045 (0.786–1.390)	0.76
	Sleep total score	-0.287	0.751 (0.563–1.002)	0.052	-0.287	0.751 (0.563–1.002)	0.052
	Somatic symptoms total score	-0.029	0.971 (0.791–1.193)	0.781	-0.029	0.971 (0.791–1.193)	0.781
	Education level 2	0.105	1.110 (0.341–3.615)	0.862	—	—	—
	Education level 3	-17.727	0.000 (0.000–.)	0.998	—	—	—

Pregnancy Outcome	Covariate	Main Model β Coefficient	Main Model OR (95%CI)	Main Model P-value	Sensitivity Model β Coefficient	Sensitivity Model OR (95%CI)	Sensitivity Model P-value
	Infertility duration	0.049	1.050 (0.903–1.220)	0.526	0.049	1.050 (0.903–1.220)	0.526

Table Notes

Model specification: Main models were adjusted for age, body mass index (BMI), infertility duration, and three-category educational attainment (reference group: junior high school and below; Education level 2 = high school/technical secondary school; Education level 3 = college and above). Sensitivity models excluded educational variables to test the robustness of results, only adjusted for age, BMI, and infertility duration. IVF = In Vitro Fertilization.

Statistical definitions: β Coefficient = Logistic regression coefficient; Odds Ratio (OR) = Exp(β); 95%CI = 95% confidence interval. P-values < 0.05 indicate statistically significant differences and are presented in bold.

Symbol explanation: Em dash (—) indicates that educational covariates were not included in the sensitivity models.

Note on OR=0.000: This value originates from SPSS iterative non-convergence, carries no clinical meaning, and is retained as raw output.
